# MICL controls inflammation in rheumatoid arthritis

**DOI:** 10.1136/annrheumdis-2014-206644

**Published:** 2015-08-14

**Authors:** Pierre Redelinghuys, Lauren Whitehead, Andrea Augello, Rebecca A Drummond, Jean-Michel Levesque, Simon Vautier, Delyth M Reid, Bernhard Kerscher, Julie A Taylor, Peter A Nigrovic, John Wright, Graeme I Murray, Janet A Willment, Lynne J Hocking, Maria J G Fernandes, Cosimo De Bari, Iain B Mcinnes, Gordon D Brown

**Affiliations:** 1 Institute of Medical Sciences, University of Aberdeen, Aberdeen, UK; 2 Faculty of Medicine, Department of Microbiology, Infectious Diseases, and Immunology, Laval University, Quebec, Canada; 3 Division of Rheumatology, Immunology and Allergy, Brigham and Women's Hospital, Boston, Massachusetts, USA; 4 Department of Orthopedic Surgery, Brigham and Women's Hospital, Boston, Massachusetts, USA; 5 Division of Applied Medicine, Department of Pathology, University of Aberdeen, Aberdeen, UK; 6 Institute of Infection, Immunity and Inflammation, University of Glasgow, Glasgow, UK

**Keywords:** Rheumatoid Arthritis, Autoimmunity, Inflammation

## Abstract

**Background:**

Myeloid inhibitory C-type lectin-like receptor (MICL, Clec12A) is a C-type lectin receptor (CLR) expressed predominantly by myeloid cells. Previous studies have suggested that MICL is involved in controlling inflammation.

**Objective:**

To determine the role of this CLR in inflammatory pathology using Clec12A^−/−^ mice.

**Methods:**

Clec12A^−/−^ mice were generated commercially and primarily characterised using the collagen antibody-induced arthritis (CAIA) model. Mechanisms and progress of disease were characterised by clinical scoring, histology, flow cytometry, irradiation bone-marrow chimera generation, administration of blocking antibodies and in vivo imaging. Characterisation of MICL in patients with rheumatoid arthritis (RA) was determined by immunohistochemistry and single nucleotide polymorphism analysis. Anti-MICL antibodies were detected in patient serum by ELISA and dot-blot analysis.

**Results:**

MICL-deficient animals did not present with pan-immune dysfunction, but exhibited markedly exacerbated inflammation during CAIA, owing to the inappropriate activation of myeloid cells. Polymorphisms of MICL were not associated with disease in patients with RA, but this CLR was the target of autoantibodies in a subset of patients with RA. In wild-type mice the administration of such antibodies recapitulated the Clec12A^−/−^ phenotype.

**Conclusions:**

MICL plays an essential role in regulating inflammation during arthritis and is an autoantigen in a subset of patients with RA. These data suggest an entirely new mechanism underlying RA pathogenesis, whereby the threshold of myeloid cell activation can be modulated by autoantibodies that bind to cell membrane-expressed inhibitory receptors.

## Introduction

The natural killer gene complex (NKC) encodes a multitude of activating and inhibitory C-type lectin receptors (CLRs), many of which play important roles in the maintenance of immune homoeostasis.^1^ Loss or mutations in inhibitory CLRs, in particular, are often associated with unchecked inflammation and destructive autoimmunity.^2^ Notably, the NKC has been associated with rheumatoid arthritis (RA),^3^ ^4^ but to date only one receptor within this cluster, dendritic cell immunoreceptor (DCIR), has been linked to this disease in humans.^3^ ^5^

Myeloid inhibitory C-type lectin-like receptor (MICL) (also known as DCAL-2, CLL-1 or KLRL1) is an inhibitory receptor found in the ‘Dectin-1 cluster’ within the NKC, whose physiological function is unknown. MICL is highly expressed on myeloid cells, particularly neutrophils, monocytes and macrophages.^6^ It possesses a single extracellular C-type lectin-like domain, that recognises endogenous ligand(s),^7^ ^8^ and a cytoplasmic tail containing an immunoreceptor tyrosine-based inhibitory motif that can transduce intracellular inhibitory signals.^8^ ^9^ MICL-mediated intracellular signalling can modulate several cellular responses,^8–11^ and expression levels of this receptor are substantially altered during cellular activation,^6^ ^7^ ^11^ suggesting that it has an immune regulatory role. Recent evidence suggests that MICL can regulate inflammation in response to cell death.^8^

There is also evidence implicating MICL in the pathogenesis of RA. We have previously described a potential link of polymorphisms in this receptor with RA pathogenesis.^12^ Moreover, expression of this receptor was shown to correlate with levels of rheumatoid factor (RF) in patients with RA.^13^ Here we have explored the physiological function of MICL and its role in RA.

## Methods

### MICL knockout mouse generation and cellular characterisation

MICL knockout mice (Clec12A^−/−^) on a C57BL/6 background were produced by Taconic Artemis, USA (see online supplementary figure S1A). *Clec12A^−/−^* and wild-type (wt) mice were bred and maintained at the Medical Research Facility, University of Aberdeen. Mice were separately housed in groups and provided freely with food and water. All experimentation conformed to the terms and conditions of UK Home Office licences for research on animals (PPL 60/4007) and the University of Aberdeen ethical review committee.

Characterisation of MICL expression in 8–12-week-old wt and Clec12A^−/−^ mice was performed by flow cytometry on cells isolated from the peripheral blood, bone marrow, peritoneal cavity, spleen and lungs, as previously described.^14^ Antibodies used in these experiments included biotin anti-MICL monoclonal antibody 309^7^ and isotype control, as well as biotin-Gr-1, FITC-7/4, PE-F480, biotin-F480, PerCpCy5.5-CD11b, PE-CCR3, biotin-NK1.1, PE-CD49, PerCpCy5.5-B220, PE-CD19, PerCpCy5.5-CD3, biotin-CD4, FITC-CD8, biotin-CD11c, PerCpCy5.5-Gr-1, APC-Ly6G, PE-CD11b, PE-Ly6G, PECy7-CD11b, Alexa Fluor 488 anti-STAT5, APC-streptavidin (all from BD Biosciences), and Alexa Fluor 700-F4/80 (BioLegend). Flow cytometry was undertaken using a BD LSRFortessa cell analyser and data analysed using FlowJo.

### Models of inflammation and infection

Mice were injected intraperitoneally (IP) with 1.0 mL Brewer's thioglycollate broth (BD Biosciences) or challenged intratracheally with either 100 ng lipopolysaccharide (LPS) or 1×10^7^ highly purified β1,3-glucan particles.^15^ Cellular inflammation was analysed by flow cytometry 24 h later, unless otherwise indicated. To characterise resistance to systemic infection, mice were injected intravenously (IV) with *Candida albicans* SC5314, and fungal burdens and survival determined as described previously.^16^ The role of MICL in adaptive immunity was explored by adoptively transferring 3×10^6^ carboxyfluoresceinsuccinimidyl ester-labelled CD45.1+ CD4+ OT.II cells into gender-matched recipient mice, which were subsequently immunised with 50 µg ovalbumin (Hyglos GmBH) in complete Freund's adjuvant (Difco). OT.II T cell responses were characterised at day 4 after immunisation by flow cytometry.

### Collagen antibody-induced arthritis

Mice were injected IV with 4 mg of ArthritoMab monoclonal antibody cocktail (MD Biosciences), and then IP 3 days later with 100 μg LPS (MD Biosciences). In some experiments, 0.7 mg of anti-MICL or isotype control antibodies were administered IP every 24 h from day 4 to day 13. Daily clinical scoring was undertaken using a scale of 0 (no visible signs of redness or swelling) to 4 (extensive swelling with signs of deformity). To generate radiation chimeras, animals received 2×500 rad (IBL437C irradiator, Cis Bio International) and were then reconstituted with 2–4×10^6^ bone marrow cells from donor mice. For histology, joints were isolated, fixed, decalcified, sectioned and stained with H&E. In vivo imaging was performed using a Bruker in vivo MS FX Pro imager, after a single IV administration of OsteoSense 680EX (NEV10020EX, PerkinElmer, UK), 24 h before the start of the collagen antibody-induced arthritis (CAIA) protocol. Twenty-four hours before image acquisition, animals were also injected IV with ProSense 750EX (NEV10001EX, PerkinElmer, UK).

### Human samples

Immunohistochemistry was performed on synovial tissue sections from patients with RA using CD163 (AbD Serotec), anti-human MICL (anti-hMICL) HB3^6^ or an IgG1 isotype control (Sigma, Oakville, Ontario, Canada). Western blotting of synoviocyte cell suspensions with anti-MICL HB3 and horseradish peroxidase-conjugated sheep anti-mouse IgG was performed using standard methodology. Anti-hMICL was detected in serum samples of controls and patients with RA by ELISA, using immobilised highly purified recombinant Fc-hMICL (see online supplementary figure S2A)^6^ or Fc-Dectin-1.^17^ Bound antibodies were detected using horseradish peroxidase-conjugated goat anti-human IgG F(ab′)_2_ fragment (Jackson ImmunoResearch). Specificity of this assay could be demonstrated by inhibition of serum antibody binding with the addition of soluble Fc-MICL (see online supplementary figure S2B). For dot blots, serum samples were used to probe recombinant histidine-tagged hMICL or histidine-tagged human C-type lectin superfamily member 8 (ClecSF8; Creative Biomart), immobilised on nitrocellulose membranes and then detected with peroxidase-conjugated goat anti-human IgG F(ab′)_2_ fragment (Jackson ImmunoResearch) or mouse anti-His monoclonal antibody (Qiagen).

Serum samples from patients with RA (see online supplementary table S3) were obtained with consent from the Institute of Infection, Immunity and Inflammation Research Tissue Bank, Gartnavel General Hospital, Glasgow, UK and with ethical approval from the West of Scotland research ethics service. Serum samples were also obtained from consenting healthy donors with the approval of the College of Life Sciences and Medicine ethics review board, University of Aberdeen.

### Statistical analysis

Two group analyses were performed using Student t tests, whereas one-way analysis of variance with Bonferroni's multiple comparison test was used for multiple comparisons. Survival was assessed by the log-rank test. All statistical analyses were performed with GraphPad Prism software V.5.04.

## Results

### MICL deficiency does not result in pan-immune regulatory dysfunction

**Figure 1:**
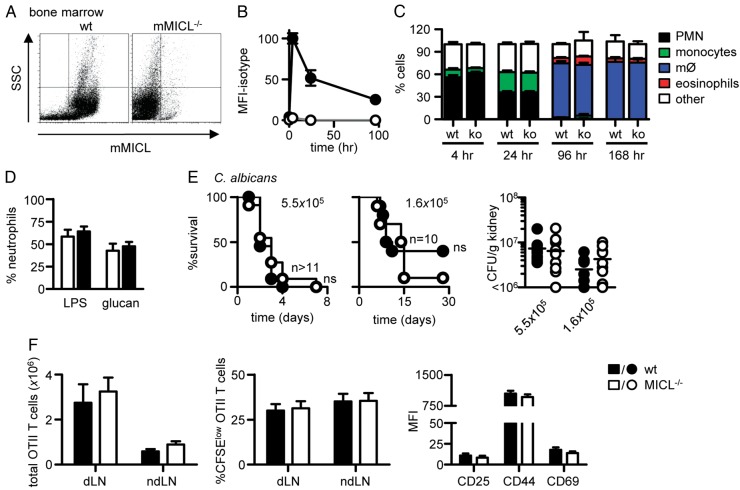
Myeloid inhibitory C-type lectin-like receptor (MICL) deficiency does not result in pan-immune dysfunction. (A) Flow cytometry analysis of MICL expression in the bone marrow of wild-type (wt) and mClec12A^−/−^ mice, as indicated. Time course analysis of (B) MICL expression and (C) cellular composition in the peritoneal cavity after thioglycollate administration. Data are pooled from at least two independent experiments and in (C) are the mean±SEM (n≥10). (D) Pulmonary neutrophil recruitment 24 h after the administration of LPS or glucan particles, as indicated. Data are pooled from two independent experiments (n=8) and expressed as mean±SEM. (E) Loss of MICL does not affect survival or kidney fungal burdens during systemic infection with *Candida albicans* (n≥10). (F) Loss of MICL does not affect the development of adaptive immunity. Characterisation of the number, division and activation of adoptively transferred OT.II T-cells in the draining (d) and non-draining (nd) lymph nodes of wild-type (wt) and Clec12A^−/−^ animals, 4 days after immunisation with ovalbumin and complete Freund's adjuvant. Shown are pooled data from at least two independent experiments (n>10). CFU, colony-forming units; CFSE, carboxyfluoresceinsuccinimidyl ester; ko, knockout; ns, not significant; LPS, lipopolysaccharide; MFI, mean fluorescence intensity; PMN, polymorphonuclear leukocyte; SSC, side scatter.

To explore a possible immune regulatory role for MICL, we generated Clec12A^−/−^ mice using conventional gene targeting, and confirmed the deletion of exons 1–4 of the gene encoding this receptor (Clec12A), corresponding to the cytoplasmic tail, transmembrane, stalk and part of the C-type lectin domain, by Southern blot and PCR analyses (see online supplementary figure S1 and data not shown). Flow cytometry of cells and tissues confirmed that MICL expression was abrogated (figure 1A and see online supplementary figure S3). The MICL-knockout mice were viable and had normal bone marrow (see online supplementary table S1) and peripheral blood leucocyte counts (see online supplementary table S2). Notably, unlike other inhibitory CLRs, including DCIR,^18^ Clec12A^−/−^ mice did not exhibit any gross abnormalities during the first 12 months of life (data not shown), suggesting that loss of MICL does not result in the spontaneous development of autoimmunity.

To evaluate immune regulatory functionality, we first explored the effect of MICL deficiency in a model of sterile peritonitis induced by thioglycollate. After in vivo challenge, peritoneal expression of MICL on leucocyte subsets reached a peak at 4 h and then slowly decreased over time (figure 1B). Characterisation of the composition of the cellular infiltrates in the Clec12A^−/−^ mice at several time points did not, however, reveal any defects in inflammation (figure 1C). Moreover, MICL deficiency had no effect on peritoneal inflammation induced by several other stimuli (see online supplementary figure S4). In addition, the knockout mice did not exhibit any abnormalities in pulmonary inflammation induced by various agonists (figure 1D and see online supplementary figure S4), or resistance to systemic infection, using a fungal infection model (figure 1E). Although MICL has been shown to regulate dendritic cell function and influence the development of adaptive immunity,^10^ ^19^ we did not detect any differences in the number, division or activation of antigen-specific CD4+ T cells in the draining lymph nodes of immunised Clec12A^−/−^ mice (figure 1F). Consistent with previous reports using another knockout mouse line,^8^ we concluded that Clec12A^−/−^ mice do not exhibit pan-immune regulatory dysfunction.

### MICL is required to control inflammation in murine models of collagen antibody-induced arthritis

**Figure 2:**
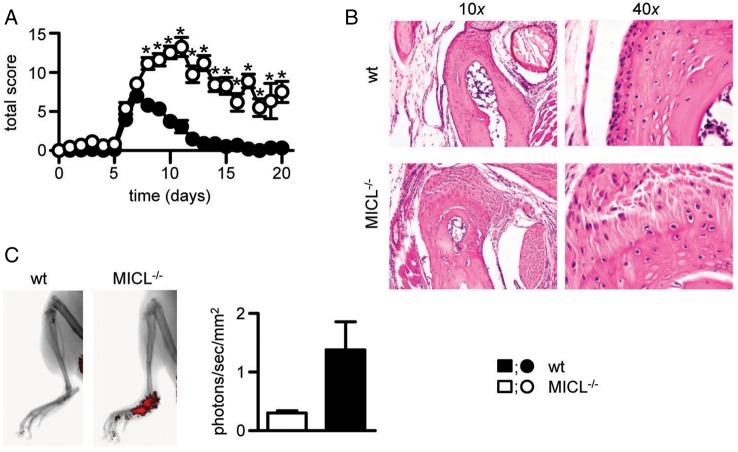
Exacerbation of collagen antibody-induced arthritis (CAIA) in Clec12A^−/−^ mice. (A) Severity scores over time during CAIA. Shown are the mean±SEM of pooled data from six independent experiments (n>42). *p<0.05. (B) Representative histopathology of the ankle joints at day 10 after immunisation. (C) In vivo imaging and measurement of inflammation during CAIA at day 17 after immunisation with ProSense (n=6). MICL, myeloid inhibitory C-type lectin-like receptor; wt, wild-type.

We next examined the effects of MICL deficiency during CAIA. This model was chosen as it induces an inflammatory polyarthritis reminiscent of the human disease and works efficiently on the C57BL/6 background.^20^ In contrast to our results described above, we elicited substantial regulatory deficit during CAIA in Clec12A^−/−^ mice (figure 2A). The peak of disease occurred around day 7 in wt mice and subsequently resolved around day 13. In the Clec12A^−/−^ mice disease severity was enhanced, with a delayed zenith response (till day 11) and failed to resolve such that, by day 20, animals still exhibited considerable articular inflammation (figure 2A). Histological analysis showed mixed leucocytic inflammatory infiltrates and signs of periarticular bone erosion in the joints of the knockout mice (figure 2B). In vivo imaging revealed increased bone remodelling (see online supplementary figure S5) and inflammation (figure 2C) in the MICL-deficient animals. Together, these data suggest that MICL plays a fundamental role in controlling the pathology of CAIA.

**Figure 3:**
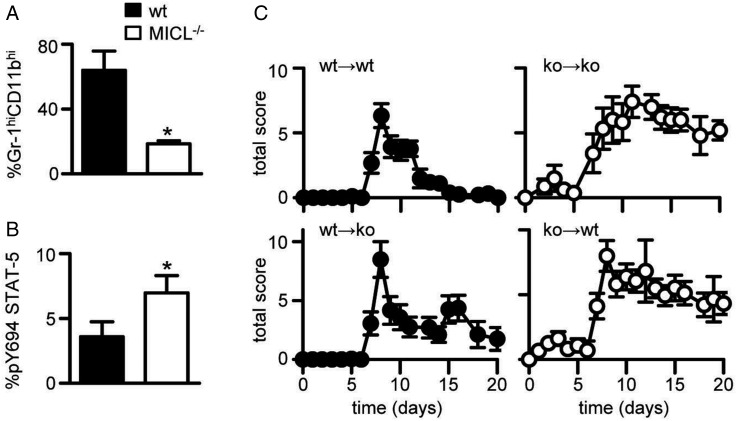
Exacerbated collagen antibody-induced arthritis (CAIA) in Clec12A^−/−^ mice stems from inappropriate myeloid cell activation. (A) Percentage of Gr-1^hi^CD11b^hi^ neutrophils and (B) expression of phosphorylated Stat-5 (pY694) at day 10 after immunisation. Data are shown as mean±SD. (C) Development of CAIA in reconstituted irradiated bone marrow chimeras, as indicated (ko, knockout; wt, wild-type). Shown are mean±SEM of pooled data from four independent experiments (n=8–24). *p<0.05. MICL, myeloid inhibitory C-type lectin-like receptor.

To gain mechanistic insight, we characterised the peripheral blood of wild-type and MICL-deficient mice during CAIA. We observed a significant reduction in Gr-1^hi^CD11b^hi^ neutrophils in the Clec12A^−/−^ mice, consistent with the increased inflammation seen in these animals (figure 3A and online supplementary figure S6). In line with the loss of an inhibitory receptor,^9^ MICL-deficient cells expressed increased phosphorylated STAT-5 during CAIA (figure 3B), indicating enhanced myeloid cell activation,^9^ a phenotype similar to that seen in mice lacking the related receptor, DCIR.^18^

To confirm that the phenotype of the Clec12A^−/−^ mice stemmed from the myeloid compartment, we generated irradiation-derived bone marrow chimeric mice (figure 3C and see online supplementary figure S7). Reconstitution of wild-type or Clec12A^−/−^ mice with MICL-knockout bone marrow exacerbated CAIA and prevented resolution of the disease, as before (figure 3C, right panels). In contrast, there was a reversion to normal progression and resolution of CAIA in Clec12A^−/−^ animals that had received wild-type bone marrow (figure 3C, left panels). These data demonstrate that the phenotype of Clec12A^−/−^ mice during CAIA stems from inappropriate regulation of myeloid cells.

### MICL is an autoantigen in a subset of patients with RA

**Figure 4:**
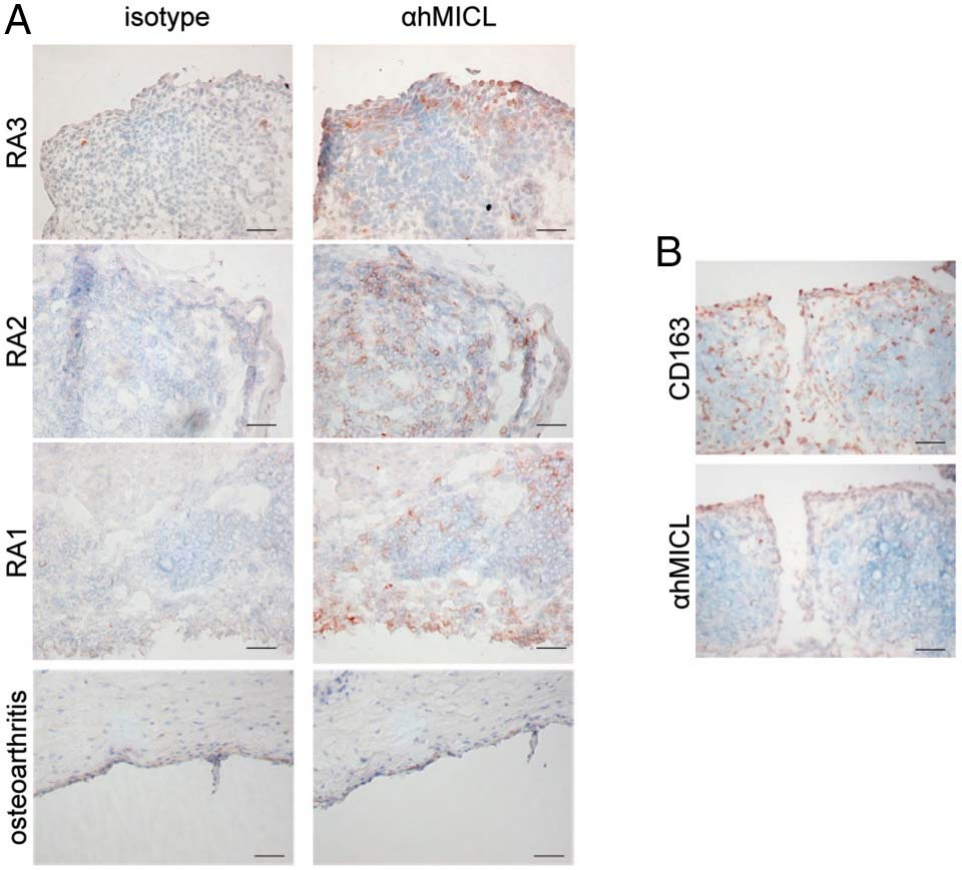
Myeloid inhibitory C-type lectin-like receptor (MICL) is expressed on myeloid cells during human rheumatoid arthritis (RA). (A) Immunohistochemical analysis of expression of hMICL (brown) in haematoxylin-stained synovial tissue sections from three patients with RA (top panels) and non-inflamed synovium from a patient with osteoarthritis (bottom panels). (B) Immunohistochemical analysis of serial sections of RA synovium probed for CD163 or αhMICL, as indicated. Sections were counterstained with haematoxylin. Scale bar, 50 μm.

To translate the relevance of these observations to human disease, we first examined the expression of hMICL in the synovium of patients with RA and controls, using monoclonal antibodies which are specific for human MICL.^6^ Immunohistochemical analysis disclosed the presence of hMICL in the synovial lining layer and in scattered subsynovial cells of patients with RA, but that there was little or no expression in non-inflamed synovium (figure 4A). Western blotting of RA synovial cell lysates further confirmed expression of the various glycoforms of MICL^6^ (see online supplementary figure S8). Staining of serial sections with CD163 suggested that MICL was expressed on cells of the macrophage/monocytic linage in inflamed synovial tissue (figure 4B).

**Figure 5:**
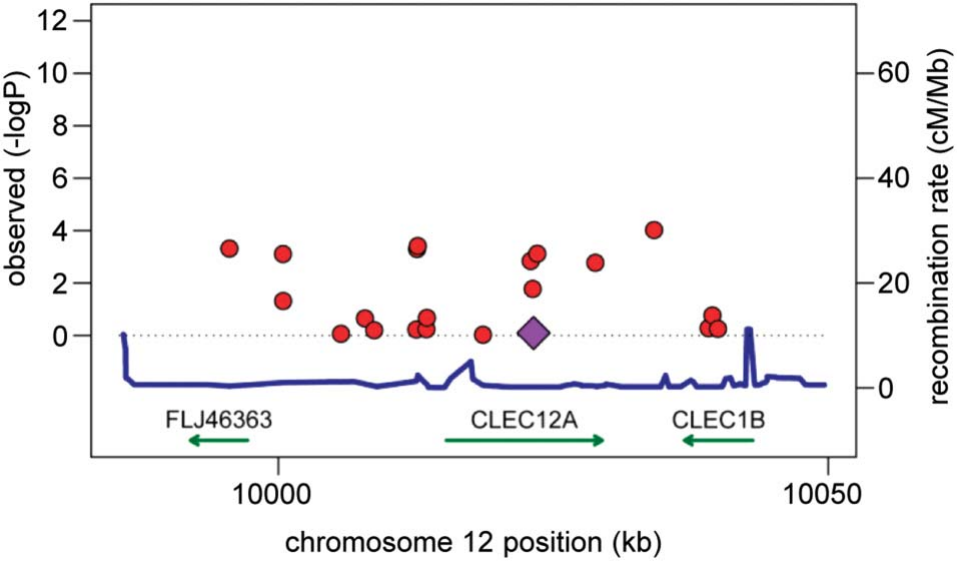
Association between single nucleotide polymorphisms (SNPs) near Clec12A and rheumatoid arthritis (RA) in the Wellcome Trust Case Control Consortium (WTCC). Tests of genetic association with RA were performed as part of the WTCC.^21^ For SNPs in and around the Clec12A locus, the −log10 (p value) from each association test is plotted against SNP chromosomal location (red dot). The purple diamond indicates rs536957 (p=0.81; OR=0.99 (95% CI 0.88 to 1.10)), which is perfectly correlated in this dataset with rs1323461, the Clec12A variant previously associated with RA.^12^ Recombination rates across the region are given by the blue line; and RefSeq gene locations are indicated by green arrows. No variants in this region achieved genome-wide significance.

The gene cluster in which MICL is located has been linked with RA,^3^ ^5^ and we recently reported that polymorphisms of hMICL could potentially be associated with this disease.^12^ We reassessed the contribution of this gene within the much larger cohort of the Wellcome Trust Case Control Consortium.^21^ However, there was no genome-wide significant association of any polymorphism in and around Clec12A with RA (figure 5).

**Figure 6:**
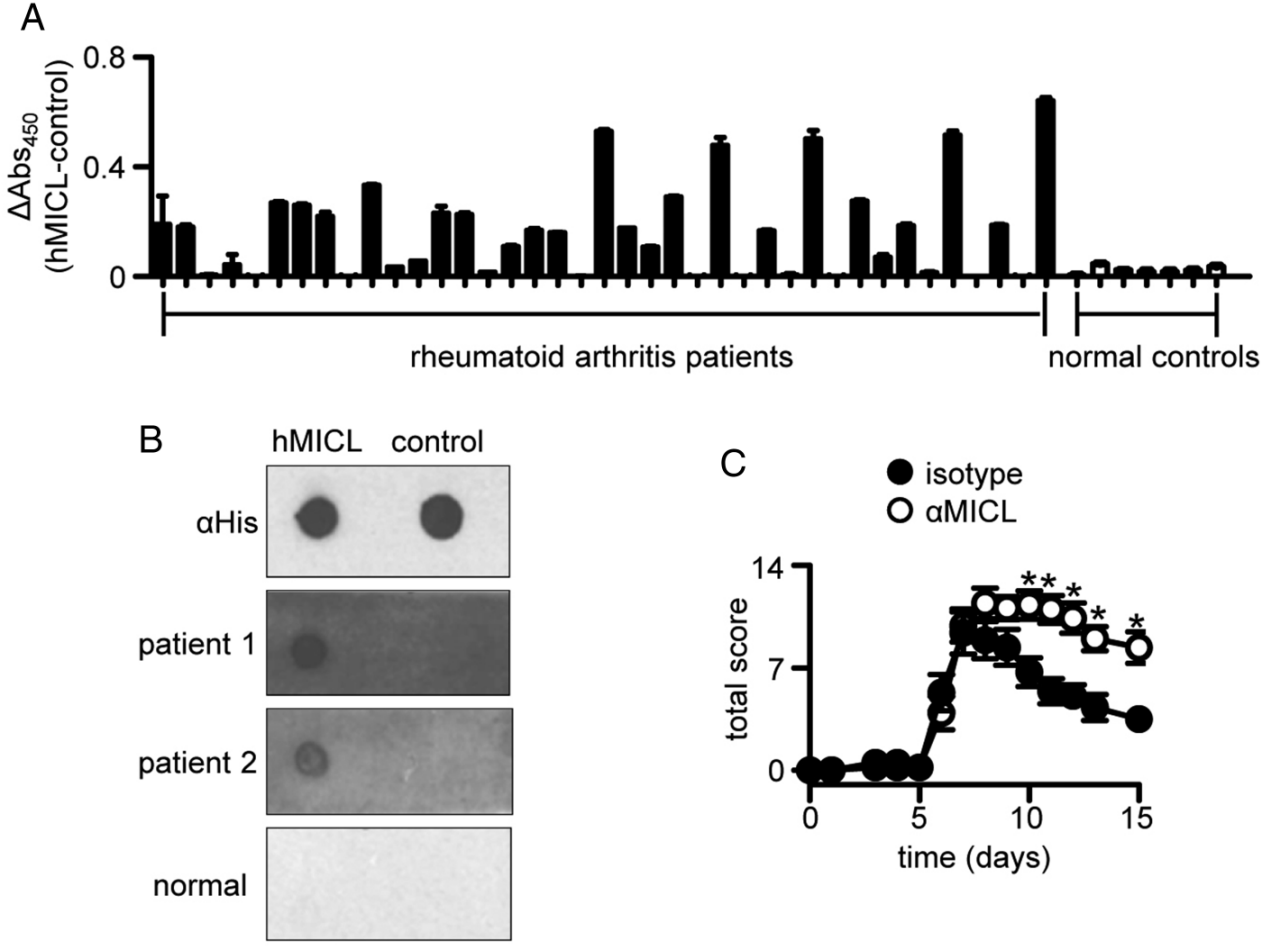
Myeloid inhibitory C-type lectin-like receptor (MICL) is an autoantigen in human rheumatoid arthritis (RA). (A) ELISA-based analyses of anti-hMICL reactivity in serum samples from patients with RA and healthy donors. (B) Dot-blot analysis of reactivity to his-tagged MICL (hMICL) and control proteins in two patients with RA and a normal control. (C) Exacerbation of collagen antibody-induced arthritis in wild-type mice after the intraperitoneal administration of anti-mMICL and isotype control monoclonal antibodies, as described in ‘Methods’. Shown are pooled data from two independent experiments (n=10). *p<0.05.

Expression of hMICL has also been correlated with raised levels of RF,^13^ so we next explored the possibility that hMICL was a target of autoantibodies in patients with RA. Notably, we could demonstrate serum reactivity to hMICL in more than 60% of patients with RA tested by ELISA, and no reactivity in healthy controls (figure 6A). These serum samples were selected at random from a small cohort of RF-positive and anti-citrullinated protein antibody (APCA)-positive patients with RA (see online supplementary table S3). To confirm this observation by an alternative method, we also directly demonstrated this serum reactivity by dot-blot analysis under native conditions (figure 6B). Serum reactivity was abolished under non-native conditions (see online supplementary figure S9). We did not detect any correlation between serum antibody levels and C-reactive protein levels or 28-joint count Disease Activity Scores in these patients (see online supplementary figure S10). Thus, these data demonstrate that a subset of patients with RA develop autoantibodies to MICL.

To determine whether anti-MICL antibodies could functionally influence the progression of arthritis, we turned to our animal models. For these experiments, we administered anti-mMICL monoclonal antibodies IP every 24 h from day 4 to day 13, after induction of CAIA in wild-type mice. We have previously described these antibodies and shown that they are specific for murine MICL.^7^ Remarkably, administration of these monoclonal antibodies increased the severity of the disease and prolonged its duration, reminiscent of the phenotype seen in the Clec12A^−/−^ mice (figure 6C). Thus these results demonstrate that antibodies to MICL can exacerbate arthritic inflammation.

## Discussion

Myeloid cells are considered central to RA pathogenesis.^22^ In this study we show that MICL can directly regulate the activation of myeloid lineage cells in inflammatory articular responses. The increased pSTAT-5 levels seen in the Clec12A knockout mice, and the previously demonstrated ability of MICL to control cellular responses,^8^ ^9^ indicate that the inhibitory signalling induced by this receptor is essential to control inflammation during CAIA. These functions of MICL are probably triggered following the recognition of cell death,^8^ which occurs in the synovium during active inflammation.^22^

Although MICL is not genetically associated with RA in humans (Okada *et al*^23^ and this study), we have discovered that this receptor is the target of autoantibodies in a subset of these patients. Although our cohort size was small, the production of autoantibodies, particularly ACPA, is a key feature in RA, and they serve as important diagnostic and prognostic indicators.^22^ Recently, direct functional activities mediated by ACPA—for example, via FcR dependent pathways, and osteoclast maturation via citrullinated-vimentin recognition have attracted increasing attention.^24^ Importantly, we now demonstrate that anti-MICL antibodies can exacerbate disease, at least in murine models, suggesting a new means whereby resolution can fail and myeloid-dependent inflammation can prevail.

There is increasing realisation that failed resolution is pivotal in driving the evolution of RA from undifferentiated inflammatory arthritis. Given the central role of myeloid lineage cells in synovitis most attention has focused on activation pathways, including TLR, FcR, NLR, PAR2 and T cell-driven lectin-dependent interactions.^22^ That a deficient autoregulatory pathway may also contribute has thus far not been identified. We thus offer an entirely new mechanism underlying disease perpetuation, or failed resolution, in which the ‘threshold’ for myeloid cell activation is modulated by autoantibody to regulatory cell-surface receptors, directly influencing the severity and persistence of inflammatory synovitis. Although further work is required to confirm that these autoantibodies have functional effects, our observations have implications for the diagnosis, stratification and treatment of this disease in patients.

## Supplementary Material

Web supplement
