# Supplementary material for: MICL controls inflammation in rheumatoid arthritis
Source: Ann Rheum Dis. 2015 Aug 14;75(7):1386–91. doi: 10.1136/annrheumdis-2014-206644 (PMC4941174; doi:10.1136/annrheumdis-2014-206644)
Supplement: Web supplement [file annrheumdis-2014-206644-s1.pdf]

Supplemental Supplementary Figures

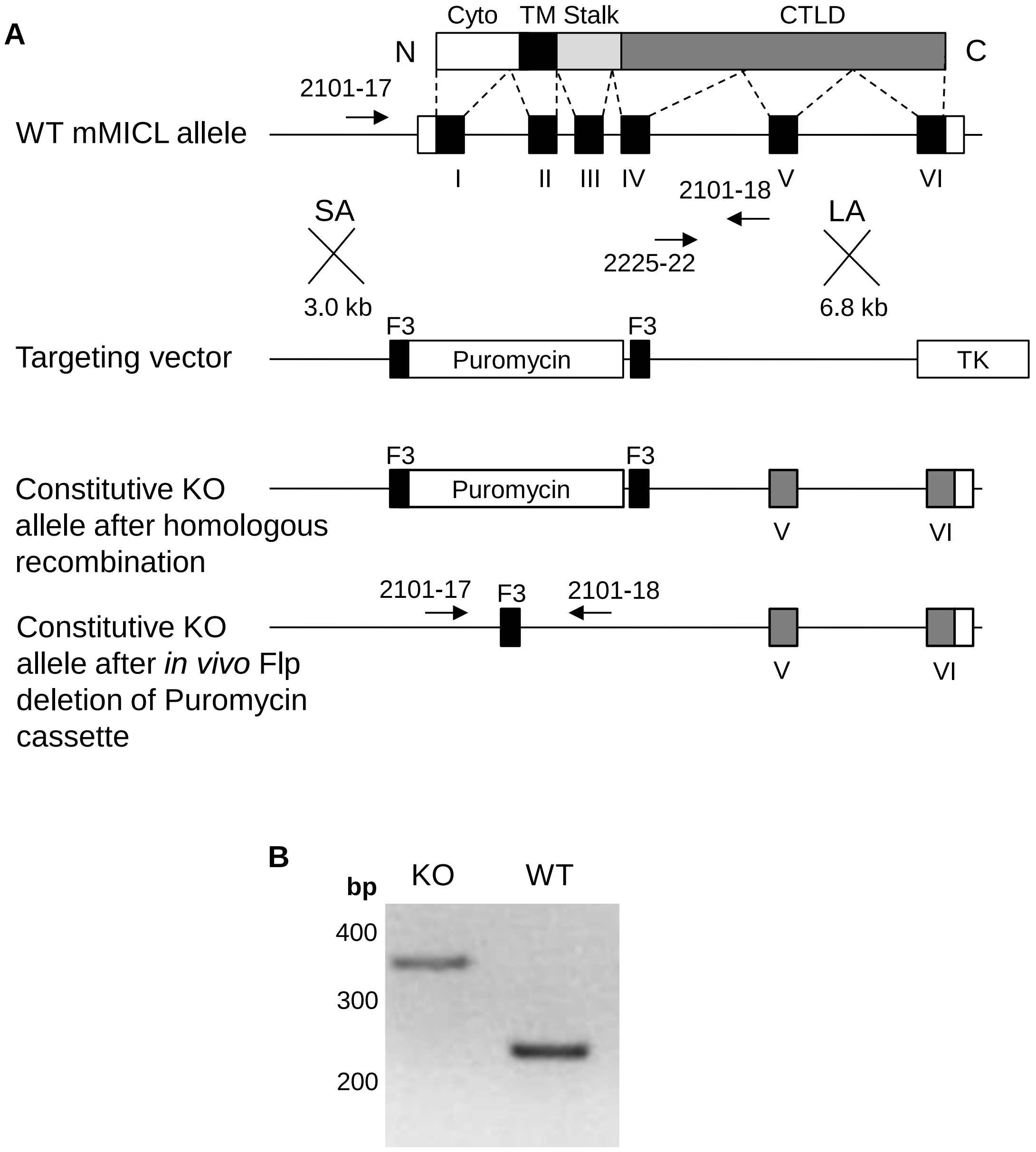

Supplementary Figure 1: Generation of the mMICL knockout mouse. (**A**) A constitutive knockout (ko) allele of murine MICL (mMICL) was generated commercially by targeting exons I-IV and a portion of the promoter region upstream of exon I, as indicated. (**B**) Mice were genotyped by PCR. Knock-out (KO) allele fragments (344bp) were amplified using the following primers: 2101-17: 5′ GATTTCCTCCTGTCCCAAGG 3′ and 2101-18: 5′ TCAACATCCACTCTGTCTTTGG 3′. Wild-type (WT) allele fragments (226bp) were amplified with 2101-18: 5′ TCAACATCCACTCTGTCTTTGG 3′ and 2225-22: 5′ GGTCTTGTACTTCATTGGACTGG 3′.

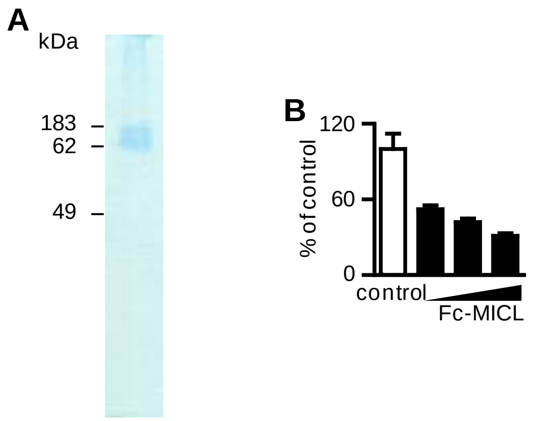

Supplementary Figure 2: Establishment of an anti-MICL antibody ELISA detection assay. (**A**) Coomassie stained SDS-PAGE gel demonstrating the purity of the Fc-MICL preparation. (**B**) Dose response inhibition of anti-MICL antibody detection in patient serum in the presence of free ligand (Fc-MICL).

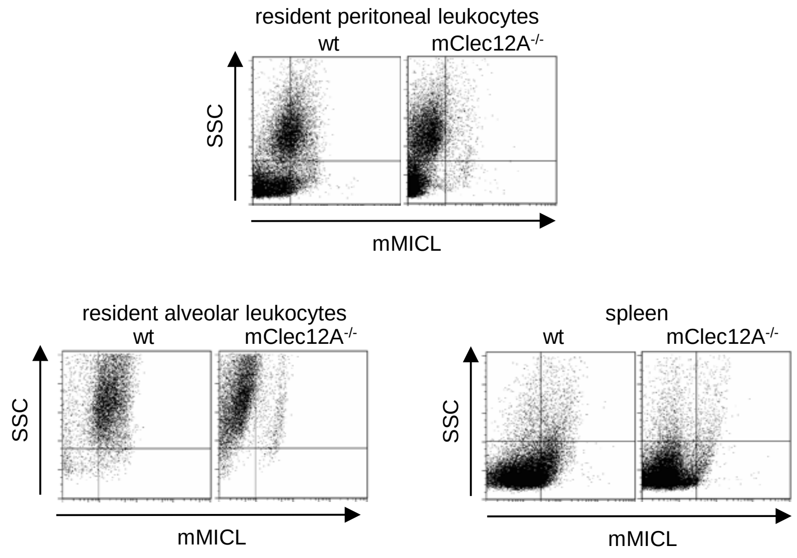

Supplementary Figure 3: Loss of MICL expression in knockout mice. Flow cytometry analysis of MICL expression on various cells and tissues of wild type (wt) and mCLEC12A$^{-/-}$ mice, as indicated

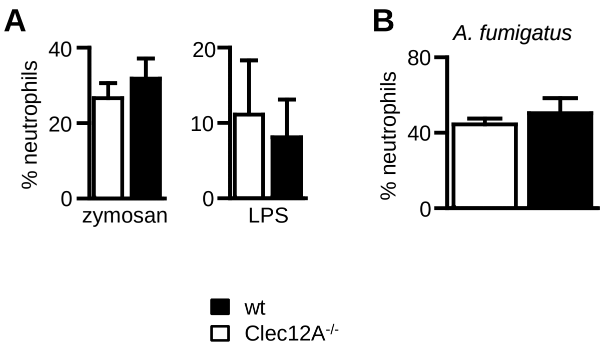

Supplementary Figure 4: Loss of MICL does not result in pan-immune dysfunction. (A) Peritoneal neutrophil recruitment 24 hr following the administration of zymosan or LPS, as indicated. Data shown are mean ± S.D (n > 4). (B) Pulmonary neutrophil inflammation 24 hr following challenge with $5x10^7$ resting *Aspergillis fumigatus* conidia. Data shown pooled from two independent experiments (n= 9 -11).

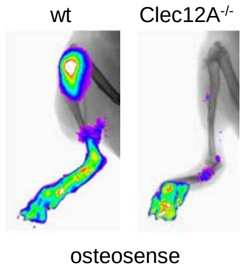

Supplementary Figure 5: *In vivo* imaging of CAIA at day 17 post immunization with OsteoSense, to measure bone remodelling, wt, wild type; ko, knockout.

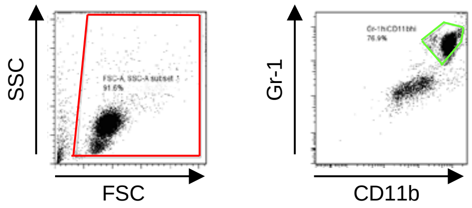

Supplementary Figure 6: Gating strategy to identify peripheral blood neutrophils. Following red blood cell lysis, cells were first gated on using FSC vs SSC (red) then on Gr-1 versus CD11b. Neutrophils (green) were identified as Gr-1^{hi} and CD11b^{hi}.

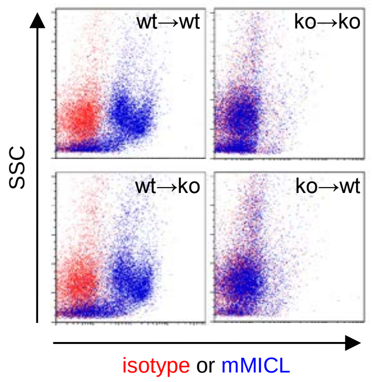

Supplementary Figure 7: Expression of MICL in irradiated bone marrow chimeric mice. Wild-type into wild-type (wt-wt), wild-type into knock-out (wt-ko), knock-out into knock-out (ko-ko) and knock-out into wild-type (ko-wt) chimeras were generated, as described in materials and methods. Bone marrow was isolated and expression of MICL analysed by flow cytometry.

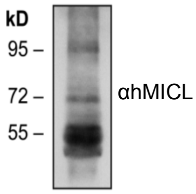

Supplementary Figure 8: Western blotting showing hMICL in RA synoviocyte cell lysates.

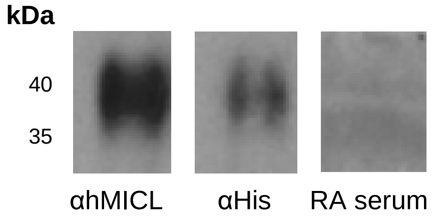

Supplementary Figure 9: Electrophoresis of hMICL under non-native conditions abolishes recognition by serum auto-antibodies. His-tagged hMICL was separated by 10% SDS-PAGE, transferred to nitrocellulose and probed with anti-hMICL, anti-his monoclonal antibody or serum from an RA patient, as indicated.

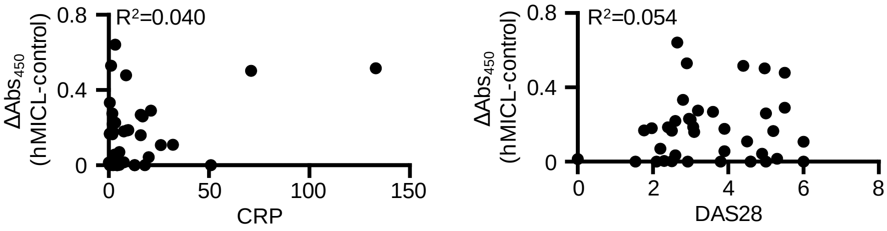

Supplementary Figure 10: Comparison of serum anti-MICL antibody levels with CRP and DAS28 scores, as indicated.

Supplementary Table 1 Differential bone marrow leukocyte counts

Cell type	Marker	Wild-type	CLEC12A$^{-/-}$
Neutrophils	Gr-1^{hi}7/4^{hi}	3.89 ± 0.53	4.30 ± 1.04
Monocytes	Gr-1^{lo}7/4^{hi}	0.68 ± 0.27	0.73 ± 0.27
	F4/80$^{hi/+}$CD11b^{+}	1.03 ± 0.05	0.98 ± 0.05
	CD11c+	0.57 ± 0.12	0.54 ± 0.16
Natural killer cells	NK1.1^{+}CD49^{+}	0.32 ± 0.05	0.31 ± 0.08
CD4^{+}T-lymphocytes	CD3^{+}CD4^{+}	0.66 ± 0.16	0.66 ± 0.13
CD8^{+}T-lymphocytes	CD3^{+}CD8^{+}	0.12 ± 0.05	0.13 ± 0.01
B-lymphocytes	B220^{+}	2.71 ± 0.85	2.70 ± 0.07

Bone marrow was isolated from matched MICL-knockout and wild-type mice and cell types were identified with the markers indicated, by flow cytometry. Data represent mean cell numbers (x10^6) ± SD and was pooled from two independent experiments (n = 8 mice).

Supplementary Table 2 Differential peripheral blood leukocyte counts

Cell type	Marker	Wild-type	CLEC12A$^{-/-}$
Neutrophils	Gr-1^{hi}7/4^{hi}	1.70 ± 0.58	1.50 ± 0.64
Monocytes	Gr-1^{lo}7/4^{hi}	1.30 ± 0.32	1.54 ± 0.58
Eosinophils	F480^{+}CD11b^{+}	3.40 ± 0.80	2.58 ± 0.47
Natural killer cells	NK1.1^{+}CD49^{+}	0.54 ± 0.04	0.54 ± 0.08
CD4^{+}T-lymphocytes	CD3^{+}CD4^{+}	1.80 ± 0.30	1.82 ± 0.35
CD8^{+}T-lymphocytes	CD3^{+}CD8^{+}	1.80 ± 0.20	1.79 ± 0.12
B-lymphocytes	B220^{+}	7.20 ± 1.60	6.57 ± 2.04

*Peripheral blood was isolated from matched MICL-knockout and wild-type mice and cell types were identified with the markers indicated, by flow cytometry. Data represent mean cell numbers (x10^{5}) ± SD and was pooled from three independent experiments representing (n > 28 mice).

Supplementary Table 3 Characteristics of the $ACPA^{+}RF^{+}$ patients

Sample	Sex	CRP	DAS28
1	F	17	5
2	F	32	4.5
3	M	1.8	5.2
4	F	0.6	1.77
5	F	1.8	2.3
6	M	2.5	2.96
7	F	5	4.9
8	F	7.6	5.3
9	M	20	4.9
10	M	4.3	2.6
11	F	7.6	1.97
12	F	16	3.6
13	F	18	5
14	M	2	2.6
15	M	16	3.1
16	F	13	4.6
17	F	4	4.6
18	F	0.6	2.8
19	F	2.9	3.9
20	F	3.2	3
21	F	26	6
22	F	21	5.5
23	F	1.9	3.2
24	F	51	6
25	F	9.1	2.4
26	F	5.4	2.2
27	F	2.2	3.9
28	F	1.2	2.9
29	F	8.6	5.5
30	M	2.1	3.8
31	F	0.5	2.5
32	F	13	2.1
33	F	71	4.97
34	F	5.4	2.5
35	F	9.8	3.07
36	F	0.5	2.93
37	F	3.2	2.65
38	F	4.3	1.54
39	F	133	4.4
40	F	ND	ND
